# Transcriptome Analysis Reveals Roles of Anthocyanin- and Jasmonic Acid-Biosynthetic Pathways in Rapeseed in Response to High Light Stress

**DOI:** 10.3390/ijms222313027

**Published:** 2021-12-01

**Authors:** Yuxiu Luo, Shoulian Teng, Hengxia Yin, Shengping Zhang, Xiaoyun Tuo, Lam-Son Phan Tran

**Affiliations:** 1 College of Eco-Environmental Engineering, Qinghai University, Xining 810016, China; 1992990033@qhu.edu.cn (Y.L.); y200713000525@qhu.edu.cn (S.T.); 1800601019@qhu.edu.cn (X.T.); 2 State Key Laboratory of Plateau Ecology and Agriculture, Qinghai University, Xining 810016, China; 3 Qinghai Academy of Agriculture and Forestry, Qinghai University, Xining 810016, China; 2019990075@qhu.edu.cn; 4 Institute of Research and Development, Duy Tan University, 03 Quang Trung, Da Nang 550000, Vietnam; 5 Institute of Genomics for Crop Abiotic Stress Tolerance, Department of Plant and Soil Science, Texas Tech University, Lubbock, TX 79409, USA

**Keywords:** anthocyanin biosynthesis, *Brassica napus*, high light, jasmonic acid pathway, transcriptome analysis

## Abstract

Rapeseed (*Brassica napus*) is one of the major important oil crops worldwide and is largely cultivated in the Qinghai-Tibetan plateau (QTP), where long and strong solar-radiation is well-known. However, the molecular mechanisms underlying rapeseed’s response to light stress are largely unknown. In the present study, the color of rapeseed seedlings changed from green to purple under high light (HL) stress conditions. Therefore, changes in anthocyanin metabolism and the transcriptome of rapeseed seedlings cultured under normal light (NL) and HL conditions were analyzed to dissect how rapeseed responds to HL at the molecular level. Results indicated that the contents of anthocyanins, especially glucosides of cyanidin, delphinidin, and petunidin, which were determined by liquid chromatography-mass spectrometry (LC-MS), increased by 9.6-, 4.2-, and 59.7-fold in rapeseed seedlings exposed to HL conditions, respectively. Next, RNA-sequencing analysis identified 7390 differentially expressed genes (DEGs), which included 4393 up-regulated and 2997 down-regulated genes. Among the up-regulated genes, many genes related to the anthocyanin-biosynthetic pathway were enriched. For example, genes encoding dihydroflavonol reductase (*BnDFR*) and anthocyanin synthase (*BnANS*) were especially induced by HL conditions, which was also confirmed by RT-qPCR analysis. In addition, two *PRODUCTION OF ANTHOCYANIN PIGMENTATION 2* (*BnPAP2*) and *GLABRA* *3* (*BnGL3*) genes encoding *MYB*-type and *bHLH*-type transcription factors, respectively, whose expression was also up-regulated by HL stress, were found to be associated with the changes in anthocyanin biosynthesis. Many genes involved in the jasmonic acid (JA)-biosynthetic pathway were also up-regulated under HL conditions. This finding, which is in agreement with the well-known positive regulatory role of JA in anthocyanin biosynthesis, suggests that the JA may also play a key role in the responses of rapeseed seedlings to HL. Collectively, these data indicate that anthocyanin biosynthesis-related and JA biosynthesis-related pathways mediate HL responses in rapeseed. These findings collectively provide mechanistic insights into the mechanisms involved in the response of rapeseed to HL stress, and the identified key genes may potentially be used to improve HL tolerance of rapeseed cultivars through genetic engineering or breeding strategies.

## 1. Introduction

Rapeseed (*Brassica napus* L., AACC, *n* = 19) is a hybrid allotetraploid species derived from *B. rapa* (AA, *n* = 10) and *B. oleracea* (CC, *n* = 9) [1]. As the third most important industrial oil crop after soybean (*Glycine max*) and palm (*Trachycarpus fortunei*) [2], rapeseed is an important source of edible oil, protein-rich animal feed, as well as biodiesel [3]. Many studies in rapeseed plants have characterized their physiological and molecular responses to abiotic stresses, such as salt [2], drought [4,5], heat [6], freezing temperatures [7], as well as cadmium stress [7,8], using transcriptome sequencing. This approach has successfully identified numerous stress response- and/or tolerance-related genes, providing information that has been dissected to better understand the underlying mechanisms associated with stress tolerance in rapeseed. To date, however, the molecular response to high light (HL) stress in rapeseed has not been fully elucidated.

As an essential environmental factor, light plays a crucial role in the growth and development of plants throughout their life cycle. However, plants will be stressful when being exposed to high intensity light, and a wide range of HL stress responses will be activated for their adaptation [9]. Previous studies mainly focused on underling photosynthetic and physiological variations, e.g., activation of non-photochemical quenching (NPQ) [10,11], changes in chloroplast and leaf avoidance movements [12,13], and leaf phenotypic changes (e.g., leaves tend to be thicken) [14]. In addition, numerous studies reported HL stress also triggered transcriptional and metabolic responses in plants [15], e.g., activation of various genes encoding enzymes responsible for reactive oxygen species (ROS) scavenging [16,17,18], and reconfiguration of primary and energy metabolism (e.g., changes in the content of various carbohydrates) [19,20,21]. However, it should be noted that the responses of plants to HL stress are diverse partly due to species-specific responses [22,23].

Anthocyanins, present in most plant species, are flavonoid secondary metabolites that serve in many cases as the source of pigmentation of a variety of plant organs, including seeds, leaves, flowers, and fruits [24,25,26]. Anthocyanins are also considered a nutraceutical and have human health-protective effects against a variety of chronic diseases, such as diabetes, allergies, viral infections, and cancer [27,28,29]. Anthocyanin accumulation in plants is frequently induced by diverse environmental stresses [30,31,32,33]. For example, anthocyanin biosynthesis is activated under HL conditions and anthocyanins function in photoprotection [34,35]. Although there have been a few studies on the roles of anthocyanin metabolism in plants under HL stress, the production of anthocyanin in rapeseed in response to HL stress has not been yet investigated.

Anthocyanins are derived from phenylalanine in a branch of the flavonoid-biosynthesis pathway. The key enzymes involved in anthocyanin biosynthesis are primarily phenylalanine ammonium lyase (PAL), cinnamic acid 4-hydroxylase (C4H), 4-coumarate-CoA ligase (4CL), chalcone synthase (CHS), chalcone isomerase (CHI), flavonoid-3′,5′-hydroxylase (F3′5′H), flavanone-3-hydroxylase (F3H), flavonoid-3′-hydroxylase (F3′H), flavonol synthase (FLS), anthocyanin reductase (ANR), dihydroflavonol reductase (DFR), anthocyanin synthase (ANS), and uridine diphosphate glucose-flavonoid- 3-*O*-glycosyltranferase (UFGT) [25,36]. Among these enzymes, DFR, ANS, ANR, and UFGT catalyze the production of anthocyanins and their glucosides in plants [36]. The predominant anthocyanins in plants are mainly derived from cyanidin, delphinidin, and petunidin [37], which may contribute to the pigmentation of plant organs [38].

It has been well documented that anthocyanin biosynthesis is primarily regulated by three types of transcription factors (TFs), namely *MYB*, *bHLH* (basic helix-loop-helix), and *WD40* that form a MBW complex, in which *MYB* TFs play a crucial role [25,39,40]. R2R3-type *MYB*s are predominantly responsible for activation of anthocyanin biosynthesis, and many *R2R3-MYB*s have been well characterized in various plant species, including *Arabidopsis thaliana* [41], grape (*Vitis vinifera*) [42], *Medicago truncatula* [43], strawberry (*Fragaria × ananassa*) [44], and apple (*Malus × domestica*) [45]. For example, *PRODUCTION OF ANTHOCYANIN PIGMENTATION 1* (*PAP1*)/*MYB75*, *PAP2*/*MYB90*, *MYB113,* and *MYB114* (*R2R3-MYB* family members) have been identified and well-studied in *Arabidopsis* [41]. *bHLH* TFs also act as important regulators in anthocyanin biosynthesis by mediating the transcriptional expression of the anthocyanin biosynthesis-associated genes *DFR*, *ANS* and *UFGT* [41]. More specifically, in *Arabidopsis,* three *bHLH* TF-encoding genes, *GLABRA 3* (*GL3*), *ENHANCER OF GLABRA 3* (*EGL3*), and *TRANSPARENT TESTA 8* (*TT8*), were demonstrated to participate in regulating anthocyanin biosynthesis [46]. Recently, the functions of *bHLH*s in regulating the anthocyanin-biosynthetic pathway have been investigated in various plant species, including apple, grape (*V. davidii*) [47], sheepgrass (*Leymus chinensis*) [48], kiwifruit (*Actinidia* spp.) [49], and lotus (*Nelumbo nucifera*) [50].

Phytohormones also play an important role in regulating plant responses to a wide variety of abiotic stresses [51]. In particular, jasmonic acid (JA) and its conjugate form, jasmonoyl-L-isoleucine (JA-Ile), have been shown to be implicated in plant responses to various abiotic stresses, such as UV, osmotic stress, drought, salinity, cold, heat, and heavy metals [52,53]. A report on the ultra-fast transcriptomic response of *Arabidopsis* to HL stress showed that ~12% of its transcripts that were induced within seconds of HL stress exposure were JA-responsive ones [54], demonstrating a potential role of this hormone in the rapid response to HL stress in plants. Additionally, JA has been reported to induce anthocyanin production in *Arabidopsis* [55,56].

In the present study, the potential roles of anthocyanins and JA in the responses of rapeseed seedlings to HL were characterized by phenotypic analysis, anthocyanin profiling, and transcriptome analysis with the main focus on anthocyanin-biosynthetic and JA-biosynthetic pathways. In addition, the expression patterns of key associated genes were validated by RT-qPCR analysis. The results of our study provide the first in-depth insight into the molecular mechanisms underlying the response of rapeseed to HL, and may potentially contribute to breeding and genetic engineering for the development of HL-tolerant rapeseed varieties.

## 2. Results

### 2.1. Changes in the Appearance and Level of Anthocyanins in Leaves of Rapeseed Seedlings in Response to HL Treatment

**Figure 1:**
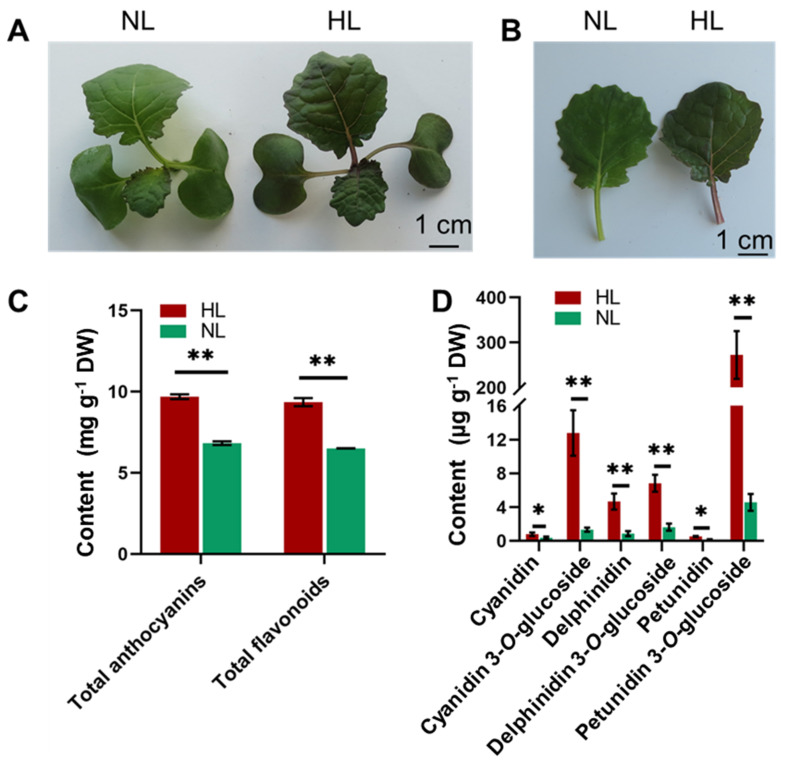
Phenotypic appearance and anthocyanin contents in rapeseed seedlings grown at different light-intensity conditions. (**A**,**B**) Colors of seedlings (**A**) and leaves (**B**) of rapeseed after 16 h exposure to normal-light (NL, 300 μmol m^−2^ s^−1^) and high-light (HL, 600 μmol m^−2^ s^−1^). (**C**) Total flavonoid and anthocyanin contents in NL and HL leaves after 16 h of exposure. (**D**) Total cyanidin, delphinidin and petunidin (three types of anthocyanins) contents in NL and HL leaves. Bars represent the means ± SDs (*n* = 3). * and ** indicate statistically significant differences between NL and HL leaves at *p* < 0.05 and *p* < 0.01, respectively, as determined by a Student’s *t*-test.

In comparison with plants exposed to a normal light (NL) intensity (300 μmol m^−2^ s^−1^), rapeseed seedlings (four-leave stage) exposed to a HL intensity (600 μmol m^−2^ s^−1^) resulted in plants with a purple appearance in the aboveground organs, including the hypocotyls, cotyledons, petioles and leaves, after 16 h of exposure (Figure 1A,B). Due to the close relationship between the pigmentation and anthocyanin accumulation in plants [57], the levels of total anthocyanins and six major anthocyanins, namely cyanidin, delphinidin, malvidin, pelargonidin, peonidin, and petunidin, in NL and HL rapeseed leaves were determined using both colorimetric assay and liquid chromatography-mass spectrometry (LC-MS). Our data revealed that the contents of total anthocyanins were 9.32 mg g^−1^ dry weight (DW) in the HL group and 6.51 mg g^−1^ DW in the NL group, indicating that a significant induction of total anthocyanins had occurred in the HL leaves (Figure 1C; Supplementary Table S1). The level of flavonoids, as precursors of anthocyanins, will reflect the potential for anthocyanin accumulation [58]. Therefore, the contents of total flavonoids in the two groups were also measured. Rapeseed leaves exposed to HL were found to have significantly higher total flavonoid contents than those exposed to NL (Figure 1C; Supplementary Table S1). Among the six anthocyanins that were measured, the levels of cyanidin, delphinidin, and petunidin were significantly higher in the HL group than in the NL group. The content of petunidin increased the most in response to HL exposure among the anthocyanins measured, exhibiting a level of 272.67 μg g^−1^ DW in the HL group, compared with 4.57 μg g^−1^ DW in leaves of the NL group (Figure 1D; Supplementary Table S1). These results indicated that anthocyanin biosynthesis was indeed involved in the response of rapeseed to HL, and that anthocyanin accumulation induced by HL might contribute to the color change in HL-exposed rapeseed seedlings.

### 2.2. Transcriptome Assembly and Analysis

Transcriptome analysis of rapeseed seedlings exposed to HL and NL for 16 h was conducted using RNA-sequencing (RNA-seq) to characterize the molecular response of rapeseed to HL intensity. A total of six cDNA libraries, representing three replicates of rapeseed seedling leaves from each light intensity group (HL group: HL1, HL2 and HL3; NL: NL1, NL2 and NL3), were constructed. High-throughput sequencing of the six libraries using the Illumina HiSeq 4000 platform generated a total of 42,937,020, 40,774,268, 42,601,798, 40,199,332, 42,171,552, and 62,116,104 raw reads for each of the six samples, respectively (Supplementary Table S2). After filtering (removal of low-quality reads and adapter sequences), approximately 6.64 GB of clean data per sample were obtained. The clean reads were aligned to the *B. napus* (PRJNA293435) reference genome using the HISAT2 software [58], resulting in an average alignment of 86% for each of the libraries, indicating that the high-throughput sequencing was of high quality. Ultimately, 198,694 genes were collectively identified from all of the samples. Furthermore, the correlation coefficient of gene expression levels among the samples (based on the fragments per kilobase per million reads) (FPKM) in each sample) was ≥0.86 (Supplementary Figure S1), indicating that the experimental samples and results were reliable and could be used for further analyses.

### 2.3. Differentially Expressed Genes (DEGs) Identified in Leaves of Rapeseed Seedlings Exposed to HL and NL Conditions

**Figure 2:**
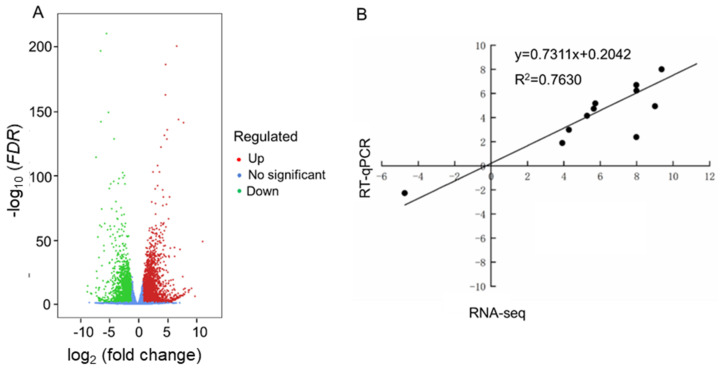
A volcano plot of differentially expressed genes (DEGs) identified in the RNA-sequencing (RNA-seq) data, and a correlation analysis of the RT-qPCR and RNA-seq data using 11 randomly-selected DEGs. (**A**) Number of DEGs in the HL/NL comparison. The *X*-axis and *Y*-axis indicate values of the log_2_ (fold change) and the −log_10_ (*FDR*) of all annotated genes in rapeseed seedlings grown under HL and NL conditions. (**B**) RT-qPCR validation of 11 DEGs. The *X*-axis and *Y*-axis represent the RT-qPCR and RNA-seq data, respectively, of the 11 randomly selected DEGs obtained from the HL/NL comparison. Data are expressed in log_2_ (fold change). HL and NL indicate high-light and normal-light samples, respectively.

A total of 7390 differentially expressed genes (DEGs), including 4393 up- and 2997 down-regulated genes, were identified in the HL/NL comparison using a threshold of |log_2_ (fold change, FC)| ≥ 1 and *FDR* (false discovery rate) < 0.05 (Figure 2A and Supplementary Table S3). Eleven DEGs shown in Supplementary Table S4 were randomly selected for the RT-qPCR analysis to validate the accuracy of the RNA-seq data. Results revealed that the relative expression levels determined by RT-qPCR were generally consistent with the transcript levels obtained by the RNA-seq data with a correlation coefficient of 0.7630 (Figure 2B), indicating that the RNA-seq data could be reliably used in further analyses.

### 2.4. Functional Enrichment Analysis of DEGs

**Figure 3:**
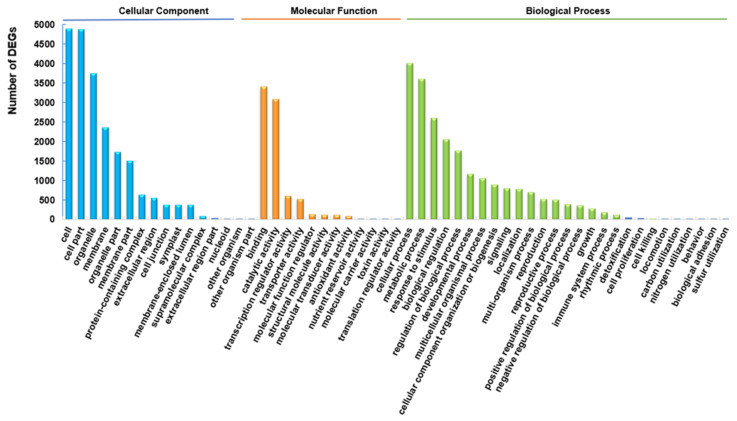
Gene ontology (GO) classification of differentially expressed genes (DEGs) obtained in the HL/NL comparison of rapeseed seedling leaves. The *X*-axis indicates the most highly-enriched 45 GO subcategories within the three primary categories. HL and NL indicate high-light and normal-light samples, respectively.

Gene ontology (GO) enrichment analysis was conducted on the DEGs identified in the HL/NL comparison to obtain information on their potential role in the responses of rapeseed seedlings to HL. Results classified the DEGs into all three of the primary GO categories: ‘Biological Process (BP)’ (27 subcategories), ‘Cellular Component (CC)’ (16 subcategories), and ‘Molecular Function (MF)’ (12 subcategories) (Figure 3). Among the BP category, the ‘cellular process’, ‘metabolic process’ and ‘response to stimulus’ were the most significantly enriched. There were more functional terms for BP, and fewer terms of CC and MF categories assigned with transcripts. The subcategories ‘cellular process’, ‘metabolic process’, and ‘response to stimulus’ in BP, ‘binding’ and ‘catalytic activity’ in MF, and ‘cell’, ‘cell part’, and ‘organelle’ in CC categories were the most highly enriched (Figure 3 and Supplementary Table S5).

Many GO terms reflected a response to light, including ‘response to light intensity’ (GO:0009642), ‘cellular response to light stimulus (GO:0071482), ‘photosynthesis, light reaction’ (GO:0019684), ‘response to high light intensity’ (GO:0009644) and ‘response to red light’ (GO:0010114) (Supplementary Table S5), which confirmed that the gene expression in rapeseed leaves was significantly influenced by HL intensity. Additionally, numerous genes were annotated to be involved in biological responses to light stimuli. For example, *BnaA03g36810D* (log_2_ (FC) = 11.30), *BnaC09g00770D* (log_2_ (FC) = 9.97), *BnaC06g20460D* (log_2_ (FC) = 9.37), *BnaCnng37300D* (log_2_ (FC) = 7.99) and *BnaA01g24440D* (log_2_ (FC) = 7.98) were all significantly up-regulated in rapeseed leaves in response to the HL treatment. Among these genes, *BnaA03g36810D,* which encodes the early light-induced protein 1 (ELIP1), was annotated in the subcategories ‘cellular response to high light intensity’ (GO:0071486), ‘response to blue light’ (GO:0071483), ‘chloroplast thylakoid membrane’ (GO:0009535), ‘photoprotection’ (GO:0010117), ‘photosynthesis’ (GO:0015979), ‘regulation of chlorophyll biosynthetic process’ (GO:0010380), and ‘photosystem I and II’ (GO:0009522 and GO:0009523) of the BP category (Supplementary Table S5). This finding strongly suggests that *BnaA03g36810D* may be involved in rapeseed responses to photosynthesis and photoprotection and may represent a potential target for manipulation to improve the tolerance of rapeseed seedlings to HL stress.

**Figure 4:**
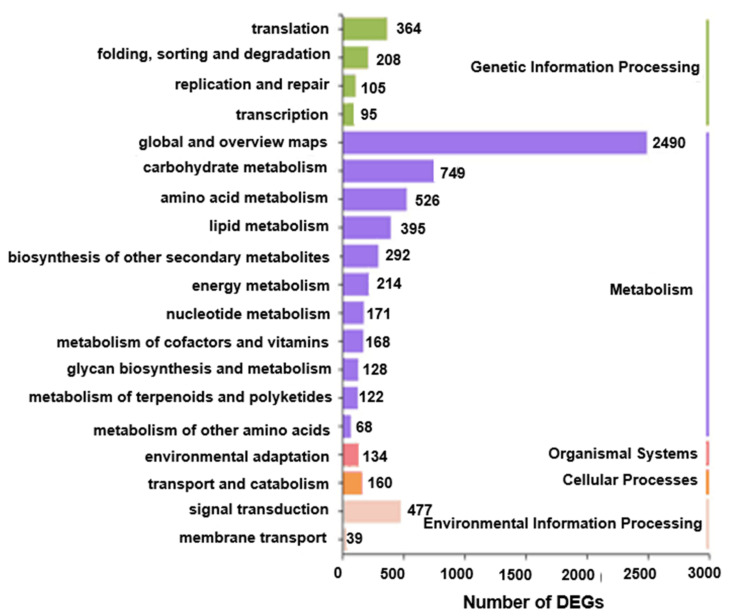
Kyoto encyclopedia of genes and genomes (KEGG) enrichment analysis of differentially expressed genes (DEGs) obtained in the HL/NL comparison of rapeseed seedling leaves. The *Y*-axis and *X*-axis represent the KEGG terms and the numbers of DEGs (HL/NL comparison), respectively. The numbers listed along each KEGG term indicate enriched DEGs found in each term. The KEGG pathways were classified into five categories indicated by different colors. HL and NL indicate high-light and normal-light samples, respectively.

Kyoto encyclopedia of genes and genomes (KEGG) enrichment analysis was performed to further understand the relationship between the identified DEGs and different aspects of metabolism. In total, five categories with 19 KEGG pathways were enriched with DEGs, namely ‘Genetic Information Processing’ (four terms, 772 DEGs), ‘Metabolism’ (11 terms, 5323 DEGs), ‘Organism Systems’ (one term, 134 DEGs), ‘Cellular Processes’ (one term, 160 DEGs) and ‘Environmental Information Processing’ (two terms, 516 DEGs), suggesting that the most enriched metabolic processes could be the main pathways involved in the responses of rapeseed seedlings to HL intensity (Figure 4 and Supplementary Table S6).

### 2.5. MapMan Analysis of Pathways Responding to HL Stress

**Figure 5:**
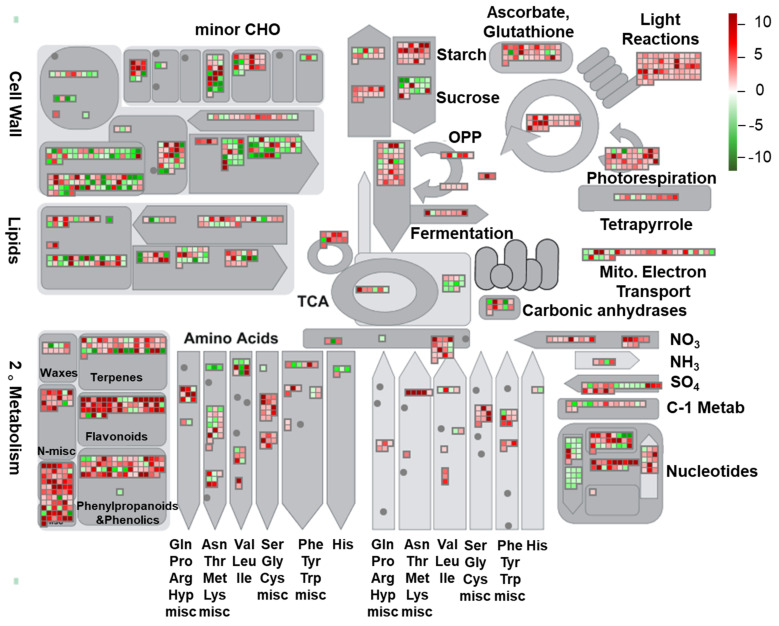
MapMan analysis of differentially expressed genes (DEGs) obtained in the HL/NL comparison of rapeseed seedling leaves, and their association with different metabolic pathways. Gray shapes indicate different metabolic pathways. The heatmaps in the gray boxes indicate up- (red-tinted boxes) or down-regulated (green-tinted boxes) genes in the HL/NL comparison. Expression changes were based on their log_2_ (fold change) values, and are presented by the colored scale. HL and NL indicate high-light and normal-light samples, respectively.

A MapMan analysis of the identified DEGs was conducted to visualize the pathways linked to the responses of rapeseed seedlings to light stress and gain more information on their biological functions. The mapping file (X4.2 *Brassica rapa*) of annotated DEGs was downloaded from the website https://mapman.gabipd.org/mapmanstore [59]. In total, 7390 DEGs were mapped to 977 pathways. The number of pathways was reduced by using the threshold of *p* < 0.05 (Figure 5; Supplementary Table S7). Among the mapped pathways, several were highly enriched (Figure 5), such as ’s-misc’, ’flavonoids’, and ‘light reaction’, indicating that these pathways may function predominantly in enabling rapeseed seedlings to tolerate HL stress.

### 2.6. Anthocyanin Biosynthesis-Related Genes Are Up-Regulated in Leaves of Rapeseed Seedlings Exposed to HL Stress

**Figure 6:**
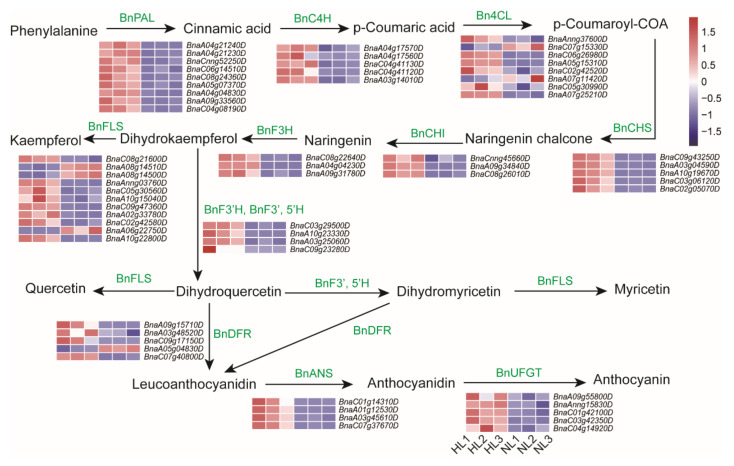
Change in the expression levels of anthocyanin biosynthesis-related genes in leaves of rapeseed seedlings exposed to high-light (HL) or normal-light (NL) intensity for 16 h. Green letters above/next to the arrows represent enzymes encoded by the indicated genes in each reaction step. The key biosynthetic enzymes are as follows: BnPAL, phenylalanine ammonium lyase; BnC4H, cinnamic acid 4-hydroxylase; Bn4CL, 4-coumarate-CoA ligase; BnCHS, chalcone synthase; BnCHI, chalcone isomerase; BnCHI, flavanone-3-hydroxylase; BnF3′H, flavonoid-3-hydroxylase; BnF3′, 5′H, flavonoid-3′,5′-hydroxylase; BnFLS, flavonol synthase; BnDFR, dihydroflavonol reductase; BnANS, anthocyanin synthase; BnANR, anthocyanin reductase; and BnUFGT, uridine diphosphate glucose-flavonoid-3-*O*-glycosyltranferase. Colored boxes and scale indicate expression levels in the HL and NL samples. Each column represents an independent biological replicate, labeled as 1, 2, and 3 following the HL or NL designation.

Since the content of total anthocyanins significantly increased in several individual plants, it is plausible that the genes participating in anthocyanin biosynthesis might exhibit a higher transcriptional abundance. Notably, the color change in plants exposed to HL was also closely correlated with anthocyanin accumulation (Figure 1). Therefore, elucidating the transcription of genes involved in anthocyanin biosynthesis will provide important information on how rapeseed responds to HL intensity. Results indicated that almost all of the anthocyanin biosynthesis-related genes were significantly up-regulated (Figure 6). In the initial stage of the general flavonoid pathway, transcripts encoding BnPAL, BnC4H, and Bn4CL were slightly up-regulated in rapeseed leaves in response to HL exposure (Figure 6). Since anthocyanin biosynthesis represents a branch of the flavonoid pathway, the biosynthesis and accumulation of flavonoids play a crucial role in anthocyanin production. All of the identified transcripts encoding BnCHS and BnCHI, which initialize flavonoid biosynthesis in rapeseed, were also significantly up-regulated. These included *BnaA03g04590D* (log_2_ (FC) = 4.94), *BnaA10g19670D* (log_2_ (FC) = 4.56) and *BnaC03g06120D* (log_2_ (FC) = 4.08) encoding BnCHS, as well as *BnaA04g04230D* (log_2_ (FC) = 5.24), *BnaC08g22640D* (log*2* (FC) = 2.51) and *BnaA09g31780D* (log_2_ (FC) = 2.38) encoding BnCHIs (Figure 6; Supplementary Table S6). In the specific anthocyanin-biosynthetic pathway, BnDFR, BnANS, and BnUFGT represent key enzymes in the production of anthocyanin [60]. Four genes encoding BnANSs, namely *Bnac07g37670D* (log_2_ (FC) = 3.87), *Bnac01g14310D* (log2 (FC) = 3.49), *Bnaa03g45610D* (log2 (FC) = 3.33), and *Bnaa01g12530D*, log_2_ (FC) = 3.11), and four BnDFR-encoding genes, including *Bnac09g17150D* (log_2_ (FC) = 6.18), *Bnaa09g15710D* (log2 (FC) = 3.62), *Bnac07g40800D* (log2 (FC) = 2.47), and *Bnaa03g48520D* (log2 (FC) = 1.37) were significantly up-regulated in response to the HL treatment (Figure 6; Supplementary Table S6). Notably, all of the BnUFGT-encoding genes, *BnaA09g55800D*, *BnaAnng15830D*, *BnaC01g42100D*, *BnaC03g42350D,* and *BnaC04g14920D*, involved in the final step of anthocyanin biosynthesis, were highly induced by HL stress, exhibiting log_2_ (FC) values ranged from 1.13 to 1.72 (Figure 6; Supplementary Table S8).

**Figure 7:**
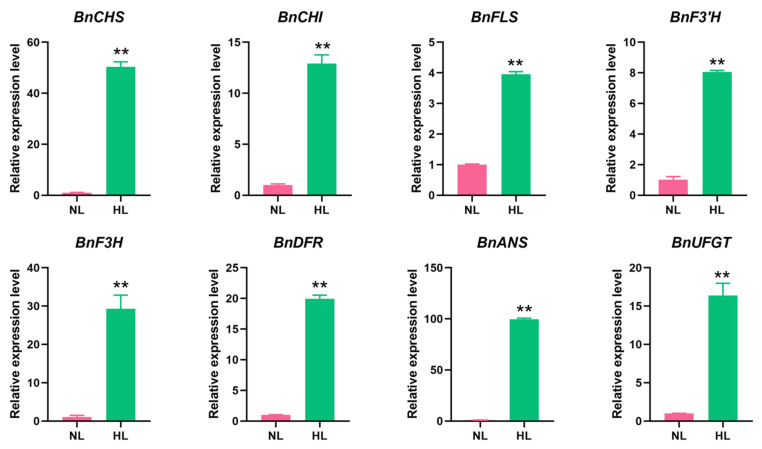
RT-qPCR analysis of 8 representative anthocyanin biosynthesis-related genes in leaves of rapeseed seedling exposed to normal-light (NL) and high-light (HL) conditions. Eight genes, namely *BnaC02g05070D*, *BnaA09g34840D*, *BnaC09g47360D*, *BnaA10g23330D*, *BnaC04g04230D*, *BnaC09g17150D*, *BnaC07g37670D* and *BnaC01g42100D* encoding BnCHS, BnCHI, BnFLS, BnF3′H, BnF3H, BnDFR, BnANS and BnUFGT, respectively, were selected. *BnACT* was used as an internal control for normalization. Data shown represent the means ± SEs (*n* = 3). ** indicates statistically significant differences between NL and HL leaves at *p* < 0.01 as determined by a Student’s *t*-test.

The expression levels of eight representative genes, namely *BnaC02g05070D*, *BnaA09g34840D*, *BnaC09g47360D*, *BnaA10g23330D*, *BnaC04g04230D*, *BnaC09g17150D*, *BnaC07g37670D,* and *BnaC01g42100D* encoding BnCHS, BnCHI, BnFLS, BnF3′H, BnF3H, BnDFR, BnANS and BnUFGT, respectively, were also studied by RT-qPCR in leaves of rapeseed seedlings exposed to HL stress (Figure 7). Notably, the relative expression level of *BnCHS*/*BnaC02g05070D* was up-regulated over 50-fold, while those of *BnANS*/*BnaC07g37670D* and *BnDFR*/*BnaC07g37670D* increased by 99.5- and 19.9-fold, respectively, after 16 h of HL exposure (Figure 7). These data strongly support the premise that anthocyanin biosynthesis and accumulation were enhanced in rapeseed seedlings in response to HL stress.

### 2.7. Analysis of Phytohormone-Related Pathways Reveals Remarkable Up-Regulation of JA Biosynthesis-Related Genes in Rapeseed Seedlings under HL Stress

**Figure 8:**
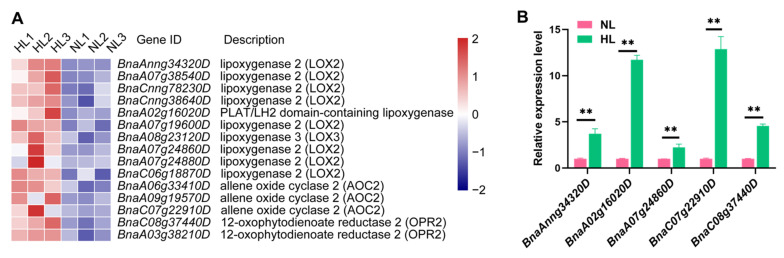
Expression and RT-qPCR analysis of JA biosynthesis-related genes in leaves of rapeseed seedlings exposed to high-light (HL) or normal-light (NL) intensity for 16 h. (**A**) Colored boxes and scale indicate normalized expression levels of all of the JA biosynthesis-related genes identified in the transcriptome data. Each column represents an independent biological replicate, labeled as 1, 2 and 3 following the HL or NL designation. (**B**) Relative expression levels of five genes related to JA-biosynthetic pathway were determined by RT-qPCR. *BnACT* was used as an internal control for normalization. Data shown represent the means ± SEs (*n* = 3). ** indicate statistically significant differences between NL and HL leaves at *p* < 0.01 as determined by a Student’s *t*-test.

Plant hormones have been demonstrated to play crucial roles in plant responses to environmental stresses [51]. In the present study, genes linked with eight different plant hormones, including auxin (IAA), abscisic acid (ABA), brassinolide (BR), ethylene (ET), cytokinin (CK), JA, salicylic acid (SA), and (gibberellin) GA, were found to respond to HL stress (Supplementary Table S9). Among these phytohormones, IAA metabolism and signal transduction pathways were associated with the greatest number of transcripts (114 transcripts), followed by the ethylene-related pathways (69 transcripts), the majority of which were down-regulated and up-regulated, respectively. Notably, all of 15 transcripts associated with JA biosynthesis identified by MapMan analysis were found to respond to HL stress and were up-regulated by HL (Figure 8A). Several studies have reported that JA mediates the biosynthesis and accumulation of anthocyanins in plants when they are exposed to adverse environmental conditions [40,42,44,45]. In our study, RT-qPCR analysis of five JA biosynthesis-associated genes also indicated that the expression of all of them was induced in rapeseed leaves by HL stress, supporting the results of the transcriptome analysis (Figure 8B). Therefore, it is plausible that JA may play a key role in plant response to HL stress and promote the accumulation of anthocyanins in rapeseed.

### 2.8. Identification of TF-Encoding Genes Involved in Anthocyanin Biosynthesis in Rapeseed

**Figure 9:**
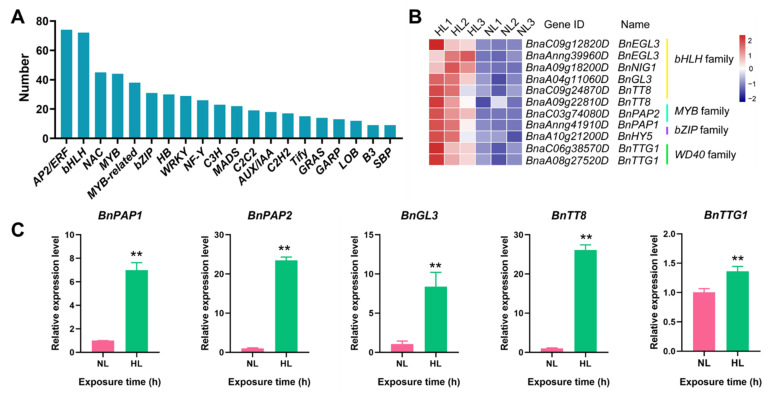
HL-responsive genes encoding transcription factors (TFs), and expression levels of HL-responsive TF-encoding genes involved in regulation of anthocyanin biosynthesis as identified in the HL/NL comparison of rapeseed seedling leaves. (**A**) The top 20 abudant TF families. (**B**) Normalized expression levels of TF-encoding genes involved in regulation of anthocyanin biosynthesis as obtained from the transcriptome data. (**C**) Relative expression levels of five selected TF-encoding genes involved in the regulation of anthocyanin biosynthesis in rapeseed seedling leaves as determined by RT-qPCR. *BnACT* was used as an internal control for normalization. Data shown represent the means ± SEs (*n* = 3). ** indicate statistically significant differences between NL and HL leaves at *p* < 0.01 as determined by a Student’s *t*-test. HL and NL indicate high-light and normal-light samples, respectively.

In the present study, 707 DEGs encoding TFs assigned to 55 different families were identified in the HL/NL comparison of leaf transcriptomes of rapeseed seedlings. A total of 448 and 259 TF-encoding DEGs were up- and down-regulated, respectively (Supplementary Table S10). The top five abundant TF families were *apetala2*/*ethylene-responsive factor* (*AP2*/*ERF*, 74), *basic helix-loop-helix* (*bHLH*, 72), *NAC* (45), *MYB* (44), and *MYB-related* (38) (Figure 9A).

Among the TF-encoding DEGs, nine genes belonging to *bHLH*, *MYB* and *bZIP* families, are known to participate in the regulation of anthocyanin biosynthesis. These anthocyanin biosynthesis-associated TF-encoding genes were significantly induced in rapeseed seedlings exposed to HL conditions, relative to the respective expression levels in seedlings exposed to NL conditions (Figure 9B). For example, members of the *bHLH* family, *BnaC09g12820D* (log_2_ (FC) = 1.67) and *BnaAnng39960D* (log_2_ (FC) = 1.84) encoding BnEGL3s, *BnaA04g11060D* (log_2_ (FC) = 2.02) encoding BnGL3, as well as *BnaC09g24870D* (log_2_ (FC) = 2.04) and *BnaA09g22810D* (log_2_ (FC) = 1.26) encoding BnTT8s were significantly up-regulated to a different extent. Two *MYB* genes, *BnaAnng41910D* and *BnaC03g74080D*, encoding BnPAP1 and BnPAP2, respectively, were significantly induced, exhibiting log_2_ (FC) values of 9.00 and 4.23, respectively, in seedlings exposed to HL conditions. Additionally, *BnaA10g21200D* (log_2_ (FC) = 1.86), belonging to the *bZIP* TF family, and encoding BnHY5, was also highly induced under HL conditions and annotated to be involved in the regulation of anthocyanin biosynthesis [61,62]. In addition, being one of the members of the MBW transcriptional complex involved in the anthocyanin-biosynthetic pathway [39], the *WD40* family of rapeseed has two members with high homology to *TRANSPARENT TESTA GLABRA 1* (*TTG1*) of *Arabidopsis*, which were identified to be differentially expressed under HL condition when compared with NL condition (Figure 9B). Specifically, the *WD40*-encoding genes *BnaC06g38570D* and *BnaA08g27520D* displayed the log_2_ (FC) values of 1.83 and 1.49, respectively, in the HL/NL comparison (Supplementary Table S3). The transcript levels of five selected TF-encoding genes, namely *BnPAP1*, *BnPAP2*, *BnGL3*, *BnTT8,* and *BnTTG1*, were also assessed by RT-qPCR, and these genes showed significant increases after 16 h of exposure to HL conditions (Figure 9C). Our analysis revealed that these 11 DEGs encoding TFs function as predominant regulators of anthocyanin biosynthesis and accumulation in rapeseed seedlings grown under HL conditions.

## 3. Discussion

Light is an essential requirement for the normal growth, development and reproduction of plants [63]. In their natural environment, plants have to continuously cope with daily and seasonal changes in light intensity. Alterations in plant color in response to light intensity represents one of the plants’ adaptive responses to light signals [64]. Rapeseed is an oil crop that is widely planted in the Qinghai-Tibetan plateau, where the solar radiation is generally stronger than that in other low-altitude environments [65]. The molecular mechanisms underlying the response of rapeseed to light intensity, however, have not been fully elucidated, which has hindered the breeding of high-quality varieties that are tolerant to HL conditions. In the present study, the observation of color changes of rapeseed seedlings in response to HL stress encouraged us to further investigate underlying mechanisms using biochemical and transcriptomic approaches.

Transcriptome analysis of leaves of rapeseed seedlings exposed to HL stress for 16 h revealed 7390 DEGs (Figure 2), indicating a considerable change in gene expression under HL stress as observed in other plant species like *Panax ginseng*, *A. thaliana* and *Begonia semperflorens* [66,67,68]. Furthermore, GO analysis demonstrated that many GO terms related to photosynthesis were most enriched with DEGs (Figure 3). The majority of identified photosynthesis-associated genes were up-regulated (Figure 5), perhaps to prevent photooxidative stress through dissipating excess light energy by NPQ or electron flow [11]. Results of KEGG analysis revealed that the pathways associated with ‘Metabolism’ were significantly enriched with DEGs, especially the primary metabolic pathways related to carbohydrates, amino acids, and lipids (Figure 4). Such changes, as also supported by results of MapMan analysis (Figure 5), might help plants meet the demand of energy metabolism, and accumulate secondary metabolites like phenylpropanoids, flavonoids, and anthocyanins that were reported to be involved in preventing the HL-induced oxidative stress through balancing the levels of ROS [16,17,19,20,69].

Numerous studies have demonstrated that a wide range of environmental stresses can stimulate the production and accumulation of anthocyanins in plants [70,71,72], suggesting that these secondary metabolites may be closely correlated with the tolerance of plants to adverse environmental conditions as indicated by earlier studies [31,33]. Although light is required for plants to generate energy for growth and development, there are potential risks in capturing light energy by the photosynthetic apparatus of plants [73,74]. Excessive light absorption under HL conditions induces photoinhibition and increases the generation and accumulation of harmful ROS, which can result in oxidative stress. Excessive levels of ROS will damage proteins, nucleic acids and membrane lipids [9,75]. Therefore, photoprotection strategies have evolved in plants to detoxify excessive ROS and maintain normal photosynthesis under HL conditions. In this regard, the accumulation of anthocyanins is a primary strategy to shield against excessive light energy through optical masking chloroplasts, as well as avoid oxidative injury by directly scavenging excessive ROS owing to the antioxidant properties of anthocyanins [76,77,78]. In the present study, the accumulation of anthocyanins and other antioxidative flavonoids significantly increased in rapeseed seedlings exposed to HL for 16 h (Figure 1C), which might reflect their ability to provide photoprotection. Notably, the level of petunidin-3-*O*-glucodise was the highest (60-fold higher than in the normal-light, control group) among the differentially accumulated anthocyanins (Figure 1D), indicating its predominant role in the color change and protective responses observed in rapeseed seedlings exposed to HL stress.

Based on these observations, analysis of the expression patterns of candidate genes involved in anthocyanin biosynthesis was conducted using RNA-seq and RT-qPCR to provide deeper insights into the molecular mechanism underlying anthocyanin accumulation in response to HL stress in rapeseed seedlings. Results indicated that the majority of genes, including biosynthesis-related and those involved in the regulation of anthocyanin biosynthesis, exhibited a rapid response and high levels of transcription in rapeseed seedlings after 16 h of exposure to HL conditions (Figure 7 and Figure 9C), resulting in enhanced anthocyanin accumulation. Numerous previous studies have reported on anthocyanin biosynthesis-associated genes and their role in the regulation of color phenotypes and tolerance to HL stress [79,80,81]. The functions of these genes have also been studied in detail using overexpression and/or silencing strategies in *Arabidopsis* or other plant species, such as apple [82], grape [42] and wheat [50]. Documenting the transcriptional expression of key anthocyanin biosynthesis-related genes may provide further insight into plant response to HL stress. In our study, the expression levels of eight representative genes *BnaC02g05070D*, *BnaA09g34840D*, *BnaC09g47360D*, *BnaA10g23330D*, *BnaC04g04230D*, *BnaC09g17150D*, *BnaC07g37670D,* and *BnaC01g42100D* encoding BnCHS, BnCHI, BnFLS, BnF3′H, BnF3H, BnDFR, BnANS, and BnUFGT were 50.4-, 12.9-, 3.95-, 8.1-, 29.3-, 19.9-, 99.5-, and 13.3-fold, respectively, higher in rapeseed seedlings under HL conditions (Figure 7). These results were consistent with previous studies, in which genes involved in the middle and late stages of anthocyanin biosynthesis were highly induced by HL stress [69,79,83]. Notably, the expression of *BnaC07g37670D* was the most induced, by 99.5-fold (Figure 7), and its encoding BnANS enzyme catalyzes the formation of colored anthocyanin precursors (anthocyanidins) [60], suggesting that this gene is of great significance in the green-to-purple color transition in rapeseed seedlings exposed to HL conditions. Additionally, the production of flavonoids, as a branch of the flavonoid-biosynthetic pathway, is crucial for anthocyanin biosynthesis [37]. The content of total flavonoids also increased dramatically in leaves of rapeseed seedlings under HL, relative to NL conditions (Figure 1C), which would contribute to the increased biosynthesis of anthocyanins (Figure 6).

*R2R3-MYB* TFs, a type of the ternary MBW complex, have been reported to play a dominant role in the regulation of anthocyanin biosynthesis [40]. Notably, *MYB75/PAP1* and *MYB90/PAP2* genes are induced by light in *Arabidopsis* [84], and their orthologs were found to be induced by 7- and 23.5-fold, respectively, in leaves of rapeseed seedlings exposed to HL conditions for 16 h in rapeseed under HL (Figure 9C) which is consistent with the observed anthocyanin accumulation under HL conditions in our study (Figure 1C,D). These data indicate that the up-regulation of both of the *PAP* genes was closely associated with the increase in anthocyanin biosynthesis and the development of purple pigmentation in rapeseed seedlings exposed to HL conditions. Similar to the color change observed in rapeseed seedlings in our study, *PAP1-* and *PAP2-*overexpressing *Arabidopsis* plants also displayed purple pigmentation in various organs, as well as anthocyanin accumulation [65]. In addition to the *R2R3-MYB*s, induction of other TF-encoding genes, such as *bHLH* and *bZIP* genes [85], in rapeseed seedlings under HL conditions, is required for anthocyanin accumulation. In our study, the transcripts of several *bHLH* TF-encoding genes, including *BnTT8*, *BnEGL3,* and *BnGL3,* that are involved in regulation of the anthocyanin biosynthesis, also significantly increased in rapeseed seedlings in response to HL stress (Figure 9C). A previous study reported that *GL3*-overexpressing *Arabidopsis* plants exhibited enhanced anthocyanin accumulation [85], indicating that *BnGL3* is involved in improving anthocyanin biosynthesis in plants in response to HL conditions. The *HY5* gene, another TF-encoding gene, whose ortholog was up-regulated in rapeseed by HL stress (Figure 9B), is well known to be involved in the light-dependent activation of anthocyanin biosynthesis-related downstream genes [86]. A recent study also reported that *HY5* plays a key role in controlling anthocyanin accumulation in plants during their response to light. For example, *HY5* is light-inducible and functions as a positive regulator of *FvbHLH9-*controlled anthocyanin accumulation in strawberry (*Fragaria × ananassa*) [61], and anthocyanin accumulation may also be positively regulated via activation of *PAP1* by HY5 in *Arabidopsis* [87]. Therefore, we propose that the *BnaA10g21200D*/*HY5,* with a log_2_ (FC) increase by 1.86 (Supplementary Table S3), is also involved in regulation of HL response and anthocyanin biosynthesis in rapeseed. In addition, the *TTG1/BnaC06g38570D* gene was also found to be induced in rapeseed leaves under HL stress. However, the induction level was lower in comparison with those of genes encoding bHLH or MYB TFs (Figure 9C). This result suggests that genes encoding *WD40* might not be as essential as those encoding *bHLH* and *MYB* TFs for the induction of anthocyanin biosynthesis under HL stress.

Numerous studies have shown that JA can positively impact anthocyanin accumulation in responses to light conditions. For example, Shan et al. reported that the molecular mechanism of JA-induced anthocyanin accumulation involves the anthocyanin biosynthesis-related regulators *PAP1*, *PAP2* and *GL3*, whose expression is induced through the activity of the JA-signaling pathway in *Arabidopsis* [87]. In our study, all of the key enzymes involved in the JA-biosynthetic pathway were up-regulated under HL stress (Figure 8A). Notably, *BnaA07g24880D* (log_2_ (FC) = 3.11), *BnaC07g22910D* (log_2_ (FC) = 3.11), and *BnaC08g37440D* (log_2_ (FC) = 1.50), which encode lipoxygenase 2 (LOX2), allene oxide cyclase 2 (AOC2), and 12-oxophytodienoate reductase 2 (OPR2), respectively, were all up-regulated in the leaves of rapeseed seedlings exposed to HL (Figure 8A), and the RT-qPCR data also support this finding (Figure 8B). Additionally, the gene *BnaC07g15660D* encoding a carboxyl methyltransferase that catalyzes the formation of methyl jasmonate (MeJA), another form of biologically active JA in addition to JA-Ile [88], was dramatically up-regulated with the log_2_ (FC) value of 4.77 (Supplementary Table S9). Previous studies have revealed that MeJA applications could enhance anthocyanin accumulation through up-regulating the transcription of related genes [89,90]. These finding together suggest that the up-regulation of JA biosynthesis by HL is required for the activation of anthocyanin biosynthesis and accumulation by modulating the genes encoding TFs (e.g., *bHLH*s and *MYB*s) that participate in the regulation of anthocyanin biosynthesis.

## 4. Materials and Methods

### 4.1. Plant Material

The inbred spring *Brassica napus* cultivar “GLH4” was used in this study. “GLH4” is an inbred line belonging to the spring-planted *Brassica napus* with characteristics of short stalk, compact plant architecture, short and upright branches, and lodging resistance. The growth period of “GLH4” is about 155 days. It begins to flower at about 60th day after germination, and the flowering period will extend to be 50 days. It is well-fitted for planting in the agricultural areas of the eastern Qinghai Province.

### 4.2. Experimental Design

“GLH4” seeds were sown in plastic pots, which were then placed in an environmental chamber (Percival LT-36VL, Perry, IA, USA) with a 16-h light photoperiod (light intensity of 300 μmol m^−2^ s^−1^) followed by 8-h of darkness. The temperatures were set at 17 °C and 4 °C during the light and dark periods, respectively. The relative humidity was maintained at 90%. When the two cotyledons were fully extended, the temperature during the dark period was changed to 10 °C, while the other conditions remained the same. When two true leaves were fully extended, seedlings of similar size were divided into two groups: NL, which served as the control, and HL which served as the treatment group. The HL group was subjected to a light intensity of 600 μmol m^−2^ s^−1^, while the light intensity of the NL group was left unchanged. After 16 h, the color change of seedlings from two groups were observed and photographed. In addition, leaves from the two groups were collected, immediately frozen in liquid nitrogen, and subsequently stored at −80 °C until they were processed for the extraction of total RNA used for transcriptome sequencing and RT-qPCR, or used in analyses of chemical constituents. Each biological replicate represented a pool of leaves collected from six plants, and three biological replicates were included for the NL and HL plants.

### 4.3. Analysis of Total Flavonoids, Total Anthocyanins and Other Anthocyanin Compounds

The extraction of total flavonoids and anthocyanins from fresh leaves of rapeseed seedlings under NL and HL conditions was carried out as previously described [64]. The contents of total flavonoids and total anthocyanins were assessed following the aluminum colorimetric procedure using the 510 and 530 nm wave lengths, respectively, with rutin being used as a standard [91].

The qualitative and quantitative analyses of 6 anthocyanin compounds, cyanidin, delphinidin, petunidin and their glucosides, were performed on a liquid chromatography-mass spectrometer (Agilent Technologies, Palo Alto, CA, USA). Purchased substances (Chengdu Herbpurity Co., Ltd., Chengdu, China) were dissolved in methanol at the 0.37–0.60 mg mL^−1^ and final concentrations were used as standards. Hence, 10 μL of each sample and standard were injected in each analysis. LC-MS analysis of anthocyanins was carried out using a reverse phase Hypersil GOLD™ C18 C18 column (150 mm × 2.1 mm, 2.6 µm, ThermoFisher, Waltham, MA, USA) with 1 mL min^−1^ flow rate over a 60-min gradient program. Further, 0.5% formic acid and acetonitrile containing 0.1% formic acid were used as solvent A and solvent B, respectively, in a linear step described previously [65].

### 4.4. RNA Isolation, Library Construction and Sequencing

Total RNA was extracted from the leaf samples using TRIzol reagent (Invitrogen, Carlsbad, CA, USA) following the manufacturer’s instructions, and the extracted RNA was subsequently treated with DNase. RNA concentration and integrity were estimated using a NanoDrop 2000 (Thermo Scientific, Waltham, MA, USA) and an Agilent 2100 BioAnalyzer (Agilent Technologies, Santa Clara, CA, USA), respectively. Enrichment of mRNA from total RNA was conducted using poly-T oligo-attached magnetic beads. The mRNA fragments were then randomly broken into short fragments that were used as a template to synthesize cDNA for construction of libraries for sequencing. The constructed six cDNA libraries including three biological replicates in NL and HL groups, respectively, were sequenced on an Illumina HiSeq 2000 platform, and paired-end reads were generated. The sequenced raw reads were submitted to the NCBI Sequence Read Archive (SRA) database (https://www.ncbi.nlm.nih.gov/sra) [92] under the SRA accession number PRJNA667589.

### 4.5. Assembly, Data Analysis and Functional Annotation

Clean reads were secured by removing adapter sequences, poly-N sequences, and low-quality (Q-value ≤ 10) reads from the raw reads. Q20, Q30, N50 and GC content values were then calculated for the clean reads. All further analyses were based on the clean data with high quality. The *B. rapa* reference genome (PRJNA293435) was used for alignment and the clean reads were mapped to the reference genome using the Hisat2 program to obtain gene annotation and position information, and the unique sequence characteristics of each of the sequenced libraries [58].

### 4.6. Identification and Functional Annotation of DEGs

FPKM for each gene were determined based on the length of the gene and read counts mapped to it. Statistical comparison of FPKM values between the two sample groups was conducted using DEseq2 software to identify DEGs using a threshold of |log_2_ (fold change)| ≥ 1 and *FDR* < 0.05 [93]. TFs were predicted by searching against the Plant Transcriptional Factor Database (http://www.plntfdb.bio.uni-potsdam.de/v3.0/) [94].

GO enrichment analysis of identified DEGs was performed using the GOseq routine in R, in which gene length bias was corrected, and GO terms with a *p*-value < 0.05 were considered as significantly enriched. KOBAS software was used to test the statistical significance of DEG enrichments in the KEGG pathways. Annotation and classification of DEGs were conducted using MapMan (http://mapman.gabipd.org/; version 3.6.0RC1) [59]. Annotated files used for MapMan analysis were downloaded from https://mapman.gabipd.org/MapManStore [59].

### 4.7. RT-qPCR Analysis

First-strand cDNA synthesis was performed with 2 μg total RNA from each replicate using the PrimeScriptRT reagent kit with gDNA Eraser (Takara, Dalian, China). The selected genes and their specific primers are provided in Supplementary Table S4. RT-qPCR was conducted in three independent biological replicates using a LightCycler 480 System (Roche, Basel, Switzerland) and TB Green Premix Ex TaqTM II (Tli RNaseH Plus) (Takara, Dalian, China). The reaction system and procedures for RT-qPCR were generally consistent with the parameters described in a previous study [63]. Relative expression levels were determined using the 2^−∆∆CT^ method [95]. *BnACT* was used as a reference gene in data analysis.

## 5. Conclusions

Light is a fundamental requirement for plant growth and development. However, excessive light capture can result in irreversible damage to chloroplasts and cell metabolism. Rapeseed is a widely grown oil crop planted in the QTP regions of China, where long-day solar and UV radiation, drought, low temperature, and low-oxygen content characterize the extreme environmental conditions. Until now, however, few studies have been conducted on the underlying molecular mechanisms involved in the response of rapeseed to HL stress. In the present study, 7390 DEGs, including 4393 up- and 2997 down-regulated genes, were identified in a transcriptome analysis of leaves of rapeseed seedlings in a HL/NL comparison. Further analyses suggested that the HL-induced DEGs were especially enriched in anthocyanin-biosynthetic and JA-biosynthetic pathways. Specially, late anthocyanin biosynthesis-related genes, such as those encoding BnANS and BnDFR, and genes involved in the regulation of anthocyanin biosynthesis, such as those encoding *BnPAP2* and *BnGL3* TFs, were highly induced by HL stress. Notably, the accumulation of anthocyanins, which may act as photoprotectants, was significantly promoted in rapeseed seedlings under HL conditions. In addition, genes related to the JA-biosynthetic pathway were also activated as part of the HL response and proposed to participate in the up-regulation of anthocyanin biosynthesis in rapeseed seedlings through JA-mediated activation of the expression of genes encoding TFs like *BnPAP1*, *BnPAP2,* and *BnGL3*. Collectively, the results of the present study provide new information pertaining to the crucial role of JA-anthocyanin biosynthesis cascade in the adaptation of rapeseed seedlings to HL stress. The insights gained from this study may help in the effort to generate HL-tolerant varieties through traditional breeding and/or genetic engineering approaches. Due to its beneficial impacts on human health, the rapeseed could be a potential source for edible anthocyanins, and rapeseed quality with high contents of diverse anthocyanins could be promoted by engineering these candidate genes involved in the anthocyanin biosynthesis.

## Data Availability

Not applicable.

## Supplementary Materials

The following are available online at https://www.mdpi.com/article/10.3390/ijms222313027/s1.
