# Supplementary material for: Transcriptome Analysis Reveals Roles of Anthocyanin- and Jasmonic Acid-Biosynthetic Pathways in Rapeseed in Response to High Light Stress
Source: Int J Mol Sci. 2021 Dec 1;22(23):13027. doi: 10.3390/ijms222313027 (PMC8657659; doi:10.3390/ijms222313027)
Supplement: Supplementary file 1 [file ijms-22-13027-s001.zip › Figure S1_202111124.pdf]

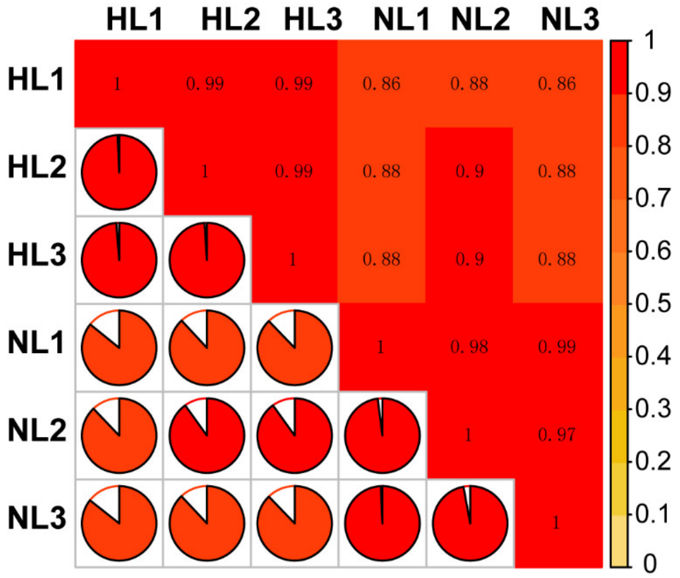

Figure S1. Pearson's correlation analysis of the samples. NL and HL indicate normal light and high light conditions, respectively. Numbers in red squares represents the correlation coefficient between samples.
